# Social determinants of health derived from people with opioid use disorder: Improving data collection, integration and use with cross-domain collaboration and reproducible, data-centric, notebook-style workflows

**DOI:** 10.3389/fmed.2023.1076794

**Published:** 2023-03-02

**Authors:** Marianthi Markatou, Oliver Kennedy, Michael Brachmann, Raktim Mukhopadhyay, Arpan Dharia, Andrew H. Talal

**Affiliations:** ^1^Department of Biostatistics (CDSE Program), University at Buffalo, Buffalo, NY, United States; ^2^Department of Medicine, Jacobs School of Medicine and Biomedical Sciences, University at Buffalo, Buffalo, NY, United States; ^3^Department of Computer Science and Engineering, University at Buffalo, Buffalo, NY, United States; ^4^Breadcrumb Analytics, Buffalo, NY, United States; ^5^Division of Gastroenterology, Hepatology and Nutrition, Jacobs School of Medicine and Biomedical Sciences, University at Buffalo, Buffalo, NY, United States

**Keywords:** clustering, cosine similarity, language model, reproducibility, social determinants of health

## Abstract

Deriving social determinants of health from underserved populations is an important step in the process of improving the well-being of these populations and in driving policy improvements to facilitate positive change in health outcomes. Collection, integration, and effective use of clinical data for this purpose presents a variety of specific challenges. We assert that combining expertise from three distinct domains, specifically, medical, statistical, and computer and data science can be applied along with provenance-aware, self-documenting workflow tools. This combination permits data integration and facilitates the creation of reproducible workflows and usable (reproducible) results from the sensitive and disparate sources of clinical data that exist for underserved populations.

## 1. Introduction

### 1.1. Motivation

Social determinants of health (SDOH) are an increasingly recognized significant contributor to health outcomes. SDOH are defined as the social, behavioral, and environmental factors that contribute to health inequalities and account for up to 70% of health outcomes (1). SDOH contribute substantially to an individual's overall physical and mental health. Specifically, low literacy, racial segregation, poverty, food insecurity, housing instability, transportation, and financial problems can impact an individual's health and contribute substantially to mortality (2). For example, place of birth is more strongly associated with life expectancy than genetics or race (1), and in the United States, a 15-year difference in life expectancy exists between the most advantaged and disadvantaged citizens (3).

In this work, we are particularly interested in SDOH as they apply to people with opioid use disorder (OUD). Substance use disorders (both illicit drug use and alcohol) affect 22.5 million individuals (2014), but only 18% received treatment (4). The indirect and direct cost of illicit drug use is estimated to be approximately USD200 billion (2007) (5). Recently, treatment of substance use disorders has emphasized harm reduction approaches and management as a chronic medical condition instead of a reliance on criminalization and incarceration (4, 6).

We are particularly interested in factors that affect treatment uptake for hepatitis C virus (HCV) infection because the infection is highly prevalent among people with OUD as injection drug use is the primary mode of transmission. HCV is a leading cause of chronic liver disease and can progress to cirrhosis, liver cancer, and death if not treated. Globally, HCV affects 58 million individuals, and HCV prevalence among people with OUD ranges from 30 to 70% (7–9). Recently, direct-acting antivirals (DAAs) against HCV have dramatically changed treatment outcomes. DAAs are all oral, curative in more than 90% of patients, and have virtually no side effects (10). DAAs have promoted the objective of HCV elimination, and interventions promoting HCV cure among people with OUD are required to achieve elimination goals (11, 12).

People with OUD are considered underserved due to limited financial resources, difficulty in accessing medical care, and underemployment. As a consequence, they typically avoid healthcare encounters in conventional medical settings due to concerns regarding stigma. As high-quality and accurate SDOH data require truthful responses from patients, investigators must consider the relationship between the patient and their healthcare provider, which is related to the trust between the patient and their healthcare provider (13, 14). Indeed, patient-provider trust is the basis of therapeutic alliances that include affective bonds, agreement on goals, and task assignment (15, 16). Patients need to have the confidence that their health information is secure, confidential, and will be protected at all times (17). When addressing SDOH among an underserved population, such as people with OUD, these factors become even more important.

A potential approach to increase the accuracy and quality of collected SDOH information may be to situate data collection in venues that people with OUD consider “safe spaces,” where they feel supported, and the environment is described as destigmatizing (18, 19). Opioid treatment programs (OTPs) have been described as accepting, comfortable, and trusting environments. The trust between patients, OTP staff, and healthcare providers largely circumvents stigma encountered in traditional healthcare settings (13, 14). Recent work has focused on the concept of health equity, that all population members should have access to high-quality health care (1, 20). Professional societies, such as the American College of Physicians (ACP), have highlighted research gaps in the area of SDOH based upon the realization that they require prioritization in order to improve health outcomes, particularly among underserved populations (1, 21, 22). Furthermore, recent data have also illustrated that SDOH are associated with geographic variation in healthcare spending, particularly in Medicare (23).

In recent years, the terms “reproducibility" and “reproducibility crisis" have been used to express concerns about research practices and selection mechanisms applied to the production and analysis of scientific data. These concerns initiated a response from the scientific community with a National Academies of Science, Engineering and Medicine (2019) report examining the issues and providing guidelines and potential solutions (24). In the field of biomedical research, Ioannidis (2005) discussed reproducibility issues in biomedical sciences. As digital medicine is seeing an explosive growth, steps need to be taken to implement the already learned lessons (25–28). This will ensure that efforts are not wasted and that the reported data and research findings are reliable. This action is particularly important if these data, and findings based upon the data, are used for formulating healthcare policy decisions. We take the term “reproducibility" to be a synonym of computational reproducibility (24, 28), which indicates the ability of a new investigator to reproduce data and results originally obtained, when the same raw materials and procedures are used.

In this paper, we will exemplify the use of computing in assembling a reproducible SDOH data set to facilitate understanding of factors that affect people with OUD pursuit of treatment for HCV infection. Our population has unique characteristics, including underemployment, being potentially stigmatized, and typically with limited financial resources, that require consideration of data collection in a safe space, which promotes accurate patient-level responses. Because a large percentage of health issues are based upon SDOH, the US federal government, in large part, is basing healthcare reimbursement through value-based payments on satisfactorily addressing SDOH. A critical research issue is how to accurately and systematically collect SDOH data, especially from underserved populations, who may be the most important target for interventions designed to improve health inequalities and outcomes. Our methods and procedures for data collection, integration, and use focus on an underserved population; however, they can be applied to all individuals in a variety of settings and have important policy implications.

### 1.2. Parent study overview

We are conducting a randomized controlled trial utilizing the stepped-wedge design at 12 OTPs throughout New York State (NYS). Telemedicine for HCV, with simultaneous administration of medications for opioid use disorder and DAAs for HCV, is being compared to offsite referral. In our study, all telemedicine encounters occurred in OTPs. Recruitment commenced in March 2017 and concluded in Feb 2020, and the study consisted of four recruitment periods of equal time length with equal numbers of participants recruited per site per period. Every site had biannual onsite staff appreciation and learning lunches with the entire OTP staff, patient advisory committee members from each site, and case managers (29).

### 1.3. Structure of the OTP

The OTP staff includes clinicians, nurses, social workers, counselors, and mental health professionals. The NYS Office of Addiction Services and Supports (OASAS) oversees a network of prevention, treatment, and recovery providers for OUD in NYS. OASAS mandates staffing ratios, frequency of in-person appearance to obtain methadone, and development of treatment plans to address OUD and its complications.

### 1.4. OASAS

Continuous engagement with NYS OASAS was critical for the implementation and conduct of the study. At the beginning of the study, we had to obtain OASAS permission to conduct telemedicine encounters in OTPs, which are under the jurisdiction of OASAS. Once permission was granted, OASAS staff assisted with recruitment of individual sites. The total number of recruited sites as well as the total number of patients recruited from each site follow the requirements and methods associated with the stepped-wedge design implementation. These are described in detail in Talal et al. (30). Furthermore, clinic interest and committment to a 5-year period was taken into consideration after we ensured clinic eligibility. We also utilized data obtained from OASAS in the following manner: 1) individual- and site-level demographic data were used in the randomization and 2) data derived from initial admission intake and annual assessments are used in the analysis of SDOH that are associated with pursuit and completion of HCV treatment through telemedicine.

### 1.5. Study purpose

One of the secondary goals of the parent study is to accurately identify the SDOH that are clinically significant and important in facilitating healthcare access, specifically HCV care access. As a first step, we seek to identify patterns of HCV care uptake as well as to understand the importance and contribution of each identified SDOH toward treatment initiation. Our population comprises individuals who uptake or decline HCV care either via telemedicine offered in the OTP or via offsite referral to a liver specialist. In this setting, we would like to identify the individual-level SDOH that differentiate the individuals who uptook treatment and/or obtained a cure and compare with those who did not.

**Figure 1:**
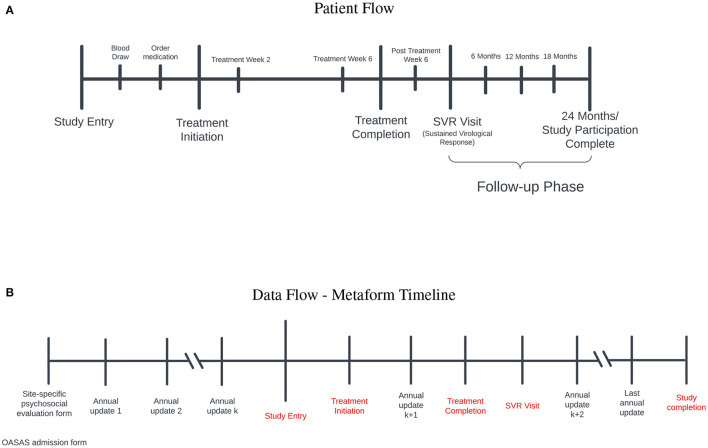
**(A)** Patient flow: Study entry occurred between 2017 and 2020. Prior to initiation of treatment for hepatitis C virus (HCV) infection, blood was obtained to ensure active infection and medications were ordered. After treatment initiation, participants were evaluated at treatment weeks 2 and 6 to ensure that the level of the virus was below detection. Once treatment was completed, participants were followed for 3 months to ensure a sustained virological response occured (SVR) (i.e., cure). Those who achieved a cure entered the follow-up phase for 2 years to ensure persistence of the cure. **(B)** Data flow-metaform timeline: Illustrated is the data flow (black) and significant study timepoints (red). Site-specific psychosocial evalaution and OASAS admission forms are collected on admission to the opioid treatment program. OASAS annual updates are completed anually. We were able to leverage these forms for several years preceding study entry to obtain a comprehensive assessment of SDOH data on each study participant. The site-specific psychosocial evaluation forms were different syntactically, and we standardized these forms for the creation of the metaform.

A timeline of significant study milestones and their relationship to SDOH data sources is illustrated in Figure 1A. SDOH were collected from a variety of sources discussed in Section 3, where the integration pipeline for the site-specific forms is also presented. These sources and methods are used to create the data set to be analyzed and are depicted in Figure 1B.

## 2. The importance of reproducibility in biomedical protocols

In 2019, the US National Academy of Sciences, Engineering and Medicine released a report on reproducibility and replicability in science, which was originated by the American Innovation and Competitiveness Act of 2017 (24).

What is reproducibility and what does it mean in different research contexts? The concept of reproducibility is complex. Reproducibility is one of the major tools science has used to establish the validity of scientific findings. It refers to obtaining consistent results using the same inputs, computational steps, methods, codes and conditions for analysis (24). As computing and data play an important role across all of science and engineering, ensuring the reproducibility of computational and data-enabled research is critical to ensure the trustworthiness of the results. Reproducibility is the minimum necessary condition for results to be believable and informative.

In our context, reproducibility means that if different investigators follow the same steps and procedures as originally described, our collection processes and methods return the same high-quality data set for analysis. This entails that our processes restrict errors in data collection that affect reproducibility. In Section 4, we elaborate on these aspects.

Two important types of errors relevant to our work are errors that produce “bad" data and errors in data management. Additional errors include errors in statistical analysis using the produced data as well as communication and logic errors. Brown et al. (31) discuss these different types of errors and their impact on scientific findings. We note here that “bad" data are data acquired through erroneous or sufficiently low quality collection methods, study designs, and/or sampling techniques.

A second type of error is associated with data management errors. These refer to errors made when handling or storing data, or when choosing a statistical method to describe or model the data. A key challenge in avoiding data management errors is the importance of context in deciding whether a particular choice (i.e., for storage or analysis of data) is an error. For example, approaches to clustering that rely on geometric means tend to perform significantly worse when applied to data sets with correlated attributes. The choice to apply k-means clustering to our data set may be reasonable, but may be considered an error on the same data set with ten additional attributes (covariates).

The issue of reproducibility of clustering results is also a well-known challenge in the relevant fields that use clustering methods [see McShane et al. (32), Dolnicar and Leisch (33), and Bollon et al. (34)]. Research on this challenge is ongoing, and validation measures seeking to evaluate the reproducibility of clusters have been developed. Kapp and Tibshirani (35) took advantage of the connection between reproducibility and prediction accuracy and developed the in group proportion (IGP) index, a validation procedure for clusters found in data sets independent of the data in which they were identified. We address this issue in two ways. First, we compute IGPs for the identified clusters; secondly, we evaluate the degree of agreement of our clustering with the PhenX dataset using cosine similarity. Section 3.3 provides a careful description of our procedures and results.

Furthermore, reproducibility also entails explainability. Knowing how and why a particular methodology was chosen for data collection, storage, or analysis is crucial for two reasons: (i) a scientist who wants to apply a comparable methodology to a new context (e.g., to apply a similar analysis to a new data set) needs to understand the reasoning behind each step of that methodology, and (ii) a scientist who identifies an interesting feature of an artifact resulting from that research methodology (e.g., a cluster of outliers on a plot) needs to be able to determine if it is a legitimate feature of the system under study, or (likely erroneously) of the methodology.

In this paper, we outline the use of a new platform for data science, named Vizier^1^ (36), that facilitates reproducibility through a combination of automated record-keeping, context tracking, and context-specific guardrails. We discuss these techniques in greater depth in Section 4. However, at a surface level, Vizier meticulously records every action that a user takes in the pursuit of a specific research artifact (e.g., a plot, model, or data set), and uses the result to build a so-called provenance graph. Choices that the user makes (e.g., casting an attribute to an integer, even if it contains non-integer values) are registered in this provenance graph, propagated through it, and presented to users as they inspect dependent artifacts. Moreover, the provenance graph is made accessible to users through several context-specific views, allowing users to quickly identify dependencies and trace specific outcomes through complicated analyses.

We also go further and outline in detail the steps taken to integrate the different data sources and to obtain a final SDOH data set to be used for understanding the impact of SDOH on an underserved population.

## 3. Data collection and integration: Challenges and solutions

### 3.1. Data sources, formats, and processes

Data for this study were collected from the following three main sources:

1. Psychosocial Evaluation forms from each site that are completed on admission (DS-1).
2. Admission Transaction Spreadsheet Report (PAS-44) and Opioid Annual Update Transaction Spreadsheet Report (PAS-26) that are completed by the site and submitted electronically to OASAS (DS-2).
3. Extracts of experimental data from the parent study, collected incrementally over the period of the study, using the MyOwnMed (37) system (DS-3).

The first data source (DS-1) consists of a range of distinct, site-specific physical forms. If a patient has multiple admissions, there are multiple psychosocial forms associated with this patient; these forms may be different syntactically and semantically from each other. In addition, data were presented in different formats, depending on the site. For example, while most sites provided paper forms, some data were provided from separate electronic health record systems and excel workbooks of evaluation questionnaires entered by site staff. Each physical form, export, or excel spreadsheet contained syntactically distinct questions and data elements and were conducted over a wide range of time from different regions across NYS.

The second data source (DS-2) was exported from OASAS's web application by each site as excel spreadsheet reports. In contrast to the high entropy of data from DS-1, DS-2 consisted of only two types of reports, each with a consistent set of data elements. Though the collection was conducted over a similar period of time as DS-1, the data elements and questions in the reports did not vary over time. The spreadsheet reports contained records from every patient of the site, not just study participants, and could only be exported 1 year at a time. If there were 30 years of records for a site, there would be thirty spreadsheet reports, each with potentially hundreds of thousands of records. Since the Institutional Review Board only allowed access to records from consented study participants, each of these reports needed to be filtered by staff at the site to contain only study participants before releasing it.

There was significant diversity, not only in the questions and data elements themselves, but with the evolution of the questions over time, and the different modalities with which the information was originally collected and maintained. The process of integrating real-world data, such as these, involved numerous methodological decisions. Recording these decisions through a tool like Vizier is critical to ensure that the resulting integrated data set can be safely re-used in new studies.

#### 3.1.1. Data collection and transfer

Psychosocial evaluation forms were located for each enrolled participant by the case manager at each site. Protected health information was redacted from each form by site staff participating in the study. In cases where the forms could be redacted on computers and saved, the files were sent securely over the internet. In contrast, paper forms were redacted and delivered physically. Regardless of how the non-structured forms were delivered, all of the data elements from each form needed to be represented as structured data. This was accomplished using two different methods. Some forms appeared in high frequency and therefore would yield more data from a single set of questions than less frequently occurring forms. These forms were represented using Javascript Object Notation (JSON) Schemas (38), and a spreadsheet entry protocol was used to represent less frequently appearing forms. Generating the JSON schema for a form is more work and is substantially more difficult technically when compared to the spreadsheet protocol, but it provides reasonable benefits, which we will describe in detail later. Team members entered data manually into either the electronic forms generated from the JSON schema or into Excel files using the protocol. Forms were reviewed for accuracy and completeness by other team members prior to submission. Specific metadata, including the submission confirmation number, entry date, entering individual, participant, and entry notes, were manually recorded in a tracking spreadsheet (manual tracker) after submission. The submissions were processed by Vizier (39), a computational notebook platform that enabled the integration, validation, and documentation of the entered data and preparation process. Vizier provides the infrastructure to automatically track and document interdependencies between preparation steps and the produced data sets. When new input data are submitted, the dependent preparation steps and output data sets can be recomputed. These, and other features of Vizier, were used to cross-reference the submissions with the manual tracker and to validate that the study participant, submission confirmation number, and submission date match the information entered in each submission, iteratively, as new data entry and submissions were ongoing. Any mismatch or discrepancy, as well as documentation provided by entry staff, is attached to each submission record and can be traced back to the source through subsequent preparation steps and transformations using the dependency graph provided by Vizier.

Particularly where data are messy, researchers are obligated to make “best-effort” attempts to wrangle the data into a form suitable for analysis. If this choice is made early in the research process, even subtle changes in analytical methodology can conflict with assumptions made during the data integration process. The same holds if the prepared data are re-used in a new analysis. Vizier's Caveats (36, 40) allows annotations on records to propagate through analyses, drawing the data scientist's attention to relevant data documentation (e.g., best-effort choices).

We worked with OASAS data management to determine what SDOH data exist within the organization and to understand methods and protocols to access it. Spreadsheet reports that are accessible by each site through an OASAS web application, specifically admission reports (PAS-44) and annual update reports (PAS-26), were adequate sources for the data of interest for the study. We developed a plan for working with the sites to assist them in acquiring the OASAS reports and in preparing the contained data to be acceptable for delivery and use in the study. The plan involved training study-supported case managers or other site staff on the process to export each report and on how to filter and prepare the data for delivery. We developed a computer application to simplify filtering, de-identification, validation and secure transmission of data. Site staff reviewed the resulting data prior to transmission. The processes employed for acquisition and delivery of the data from sites varied between data sources, but for any data that flowed over the internet, transport layer security and multi-factor authentication were used to provide a secure channel for the transmission.

#### 3.1.2. JSON Schemas and JSON schema forms for data entry

JSON Schema (38) is an Internet Engineering Task Force (IETF) standard specification for defining the structure of data that allows the annotation and validation of JSON (41, 42) documents. It provides clear human and machine-readable documentation that can help with automated validation, transformation, and quality control of client-submitted data (43). JSON Schema describes the names, data types, and properties of data elements of a JSON document and the hierarchy of those elements. Yet, it does not describe how a given data type should be rendered as a form input component. We used JSON uiSchema (44), a metadata format that captures how the elements of a JSON schema should be displayed (i.e., as a form) in a user interface. The uiSchema object follows the tree structure of the form field hierarchy and defines how each property should be displayed to the user, describing the general layout of a form by using different uiSchema elements, which can often be categorized into either Controls or Layouts. Some uiSchema elements allow an options property, allowing further configuration of the rendering result.

**Figure 2:**
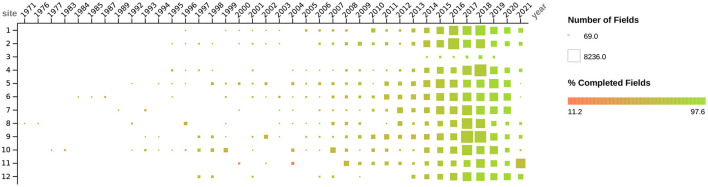
Figure depicts the dates, quantity, and degree of completeness for all social determinants of health data (i.e., DS-1 and DS-2) collected as described above by study sites. Dates when forms were administered to participants range from 1971 to 2021. The quantity of data collected (i.e., number of fields) from each site is depicted by the size of the box, ranging from 69 to 8236 fields. The color of the box, ranging from red to green, depicts the degree of field completeness, ranging from 11.2 to 97.6%.

Because of the wide variety of physical forms, the varying frequency that instances with which each form appeared, and the high degree of evolution of these forms over time, there was a motivation to efficiently translate each type of form to a simple data entry interface. We wanted to make it easy to perform data entry and enable the automation of quality checks, such as schema validation and data management of evolving schemas. We found that generating a JSON Schema that maps every form section and question to a JSON object that reflects the structure and content of the physical form sufficiently satisfies these motivations. Form sections and subsections were encoded into the schema as nested objects that matched the hierarchy of the sections and subsections in the form, and they were named matching the respective titles of those sections. Questions are included in the hierarchy where they appear in the physical form respectively and are named with the text of the question. Questions with free text answers are encoded as string fields, number questions as number fields, multiple choice questions as string or number fields with Enumerated Values (or “enums,” which restrict JSON instances to have certain values specified in the schema as an array), and multiple answer questions as array fields. The JSON Schemas with an associated JSON uiSchema were then used to render data entry forms that enforce the schema during entry and submission using React JSON Schema Forms (45), a react component for rendering JSON Schemas as web browser-based data entry forms. Forms that were entered into Excel workbooks lacked the initial schema enforcement on data entry but were preprocessed after submission to infer a JSON Schema that we then used to ingest the excel workbooks into the same workflow used to process the React JSON Schema entered physical form submissions. We reused this same process again to ingest the OASAS spreadsheet reports. The result was one data set that contained all the data from DS-1 and DS-2, for which the number of data elements over time and the percentage of those elements that were complete is summarized in Figure 2. Since all of the data except DS-3 are now in one data set, and every data element is represented in conformance to a JSON schema, irrespective of the submission from which it originated, we can walk over the schemas and automate tasks. These include secondary data validation and extraction of data elements of interest in a way that is flexible to the introduction of new data and schemas. Where errors occur, we can attach caveats (36, 40) so that the errors are noticed when the resulting data sets are used.

JSON Schemas enable a method of traversing data elements where the types and hierarchy are known, but the traversal itself is not dependent on those types or their hierarchy. This is more flexible to the introduction of new data and can improve the ability of researchers to more easily accept new data and understand how that data evolve over time.

### 3.2. Models and algorithms

#### 3.2.1. Data-centric notebook-style workflows

**Figure 3:**
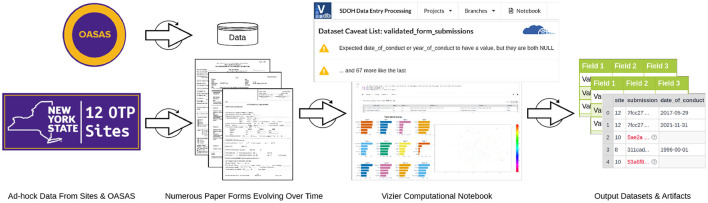
Iterative flow of data during *ad-hoc* collection and integration.

At the outset of data collection planning, we did not know the exact content of the data we would be collecting, the volume, or even the source and format. As we gained more information on the acquisition details, it became evident that the collection would be occurring incrementally, that it would be from multiple sources, and that the medium of data delivery would be disparate. With the limited resources for data collection and preparation, we needed an efficient method to bring the diverse data together that was flexible enough to handle not only the *ad-hoc* acquisition of data but also the evolving understanding of the content of that data. When data collection is *ad-hoc*, data arrive incrementally as they are available; for this study, either they were delivered from a site after extraction, or they were submitted by data entry staff one form at a time as they completed entry. Because the content of the data is unknown before it arrives in some cases, and it is coming incrementally, the preparation and processing of the data are forced to be incremental as well. As new input data become available, changes to how data are processed may be needed, or additional data may need to be added to maintain use cases of output data sets. For example, when a critical data element assumed to be present for all forms is missing from a newly submitted form, the workflow caveats the data for the investigator. In our study, the date of conduct (i.e., the date when a particular form was administered to a participant) was missing from a subset of data from two different sites. Instead of the workflow opaquely failing to complete without an explanation, or worse, completing and using incorrect default value assignments (e.g., the assignment of date of 01-01-1900 to forms missing the actual date of conduct), which is known to occur in existing ETL systems (46), Vizier caveats the data with an explanation of what went wrong and where. Figure 3 illustrates a simplified representation of the iterative, *ad-hoc* flow of information from the sources of data to the resulting output data sets. It highlights the use of caveats and how they draw attention to (the red values in the output data set) and explain (the “Dataset Caveat List") errors that can occur so that they can be addressed, like the example of missing dates of conduct. To address the error in this specific case, the missing dates of conduct had to be acquired from the site and integrated into the workflow by adding two steps or “cells,” one to ingest the newly acquired dates of conduct data and one to join those with the records missing the dates. All subsequent transformations and steps in the workflow that use the output from the previous step are re-evaluated automatically.

Adaptable workflows that can repeat previous work on new information, automatically propagating changes, are safer for use on incrementally evolving data (e.g., when integration takes place concurrently with data collection). The cognitive burden on data scientists is lower, and there is less risk that a missed processing step will leave stale data in the workflow. Moreover, the same information provides explainability, reducing the time required to track down data integration errors.

#### 3.2.2. Multi-modal and multi-lingual

Different facets of data collection and curation require different approaches, programming languages, libraries, and tools. Existing languages and libraries are often specialized for the specific details of a task. For example, JSON Schema forms and spreadsheets are ideal for data entry because high-level technical skills are not required, and they have some “guard rails,” like schema enforcement through form validation. The Python programming language has many libraries and tools for data wrangling. The Scala programming language and Spark are excellent for data processing. Structured Query Language (SQL) is designed for relational data querying. Traditionally, bringing all of these language tools together to be used in a cohesive and seamless way is a difficult and problematic undertaking in itself. However, the process often arises naturally on projects precisely for the reasons just outlined. These projects can become very complex quickly and can span multiple code files written by a variety of developers (46) with numerous dependencies that require installation and maintenance. Managing such projects can be infeasible for small research teams or organizations with limited resources. Seamless integration of these features without technical management, dependency tracking to prevent stale data, propagation of documentation, and explainability of errors through caveats reduce management complexity and can improve the focus of data scientists and researchers on the data.

### 3.3. Semantic alignment of data

#### 3.3.1. Definition of NLP models and description of semantic alignment concept

Exploring the relationships between different SDOH variables derived from the self-reported data (from DS-1 and DS-2) and outcomes in the experimental data (DS-3) was a necessary goal. To do this exploration, we first need to align time points of specific milestone events in the experimental data of the parent study for each participant with self-reported data collected nearest to those time points. Common SDOH variables need to be derived from the data elements in the self-reported data across the different and diverse sets of forms that were collected and time-aligned with the experimental data milestone events. As the data were collected and structured, each question that appeared on a form was recorded along with the form section and subsection headings. For example, the question “What Is The Highest Grade You Have Completed” that appeared on a form in a section titled “economic” and subsection titled “Education” would be recorded with a “field name” of “What Is The Highest Grade You Have Completed” and a “field path” of “economic/Education/.” On another form, the same question appeared “What Is The Highest Grade You Have Completed,” but under a section titled “Education Data", which would be recorded with a “field name” of “What Is The Highest Grade You Have Completed” and a “field path” of “Educational Data/.” These two questions ask the same thing, but appear in sections with different titles and/or subsection titles. In many instances, a revision of a form would change a section title, which results in questions being recorded with different “field paths.” We recorded 7,519 distinct questions when the section title in which a question appears and the text of the question is used to determine if a question is distinct. If we only consider the exact wording of the question itself as determining the distinctness of a question, then that reduces the number of distinct questions to 3,582. On another form, there was a question, “Highest grade attained” under a section titled “social” and, subsection “Education History.” In this case, the question's wording is different, but the question is, semantically very similar. For the purposes of this study, we would want to consider all three versions of the question in the same SDOH variable category. Grouping the questions from the approximately 49 distinct forms that have been utilized for data collection on SDOH by semantic similarity can assist in deriving the SDOH variables and exploring the relationships between SDOH variables and the experimental data milestones.

#### 3.3.2. Metaform creation

**Figure 4:**
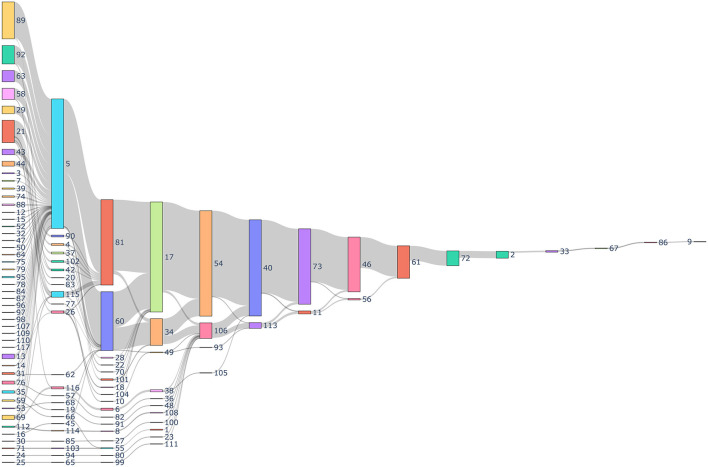
Sankey diagram depicting the history of data collection through different forms. Every number indicates a unique identification indicating the form and the sequence in which it was collected for a patient. The gray links represents the number of patients. The size of the nodes represent the number of total forms collected.

The process of identifying SDOH categories for data collection is an iterative process, which was challenging based upon the large number of forms as depicted in Figure 4. The first step of this process included using language models, dimensionality reduction, and clustering algorithms to identify the clusters. These steps facilitated labeling. The subject matter experts (SMEs) initially developed a label that best defined each cluster. At the same time, SMEs realized that some questions would benefit from being placed in a different cluster because they did not pertain to the main idea indicated by the cluster label. The process was continually refined and became more accurate as additional questions were added.

At the final step, the SMEs were provided with the clustering results and were asked to evaluate whether the assignment of each specific question to the designated cluster was correct. This exercise resulted in the SMEs identifying that at least 90% of the questions were correctly assigned to their designated cluster by the clustering algorithm. The SMEs then assigned the remaining questions to the appropriate cluster.

### 3.4. Identifying and validating SDOH categories

**Figure 5:**
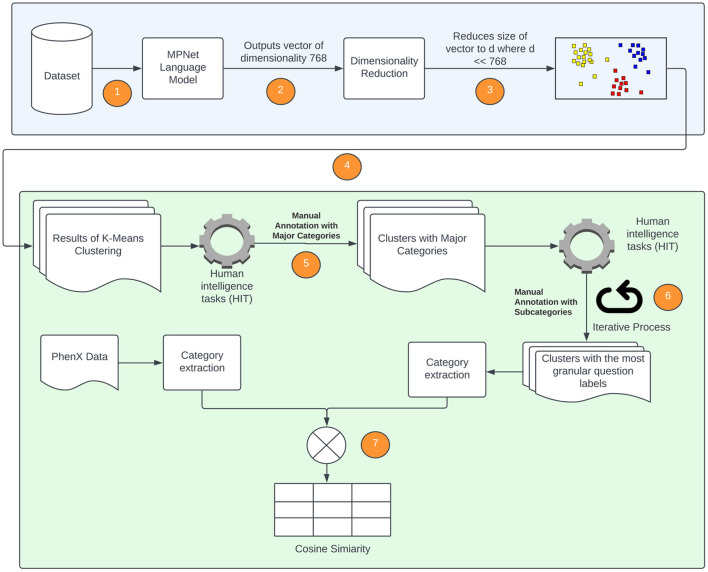
Diagrammatic representation of processes followed to extract SDOH data from available forms. The numeric steps of the SDOH extraction pipeline are incorporated, and correspond to the following. 1. The language model is applied to the forms used to extract SDOH data; 2. The language model outputs a vector of dimension 768 × 1; 3. Locally Linear Embedding (LLE) is applied to reduce the dimension; 4. K-Means (spherical) is applied to data obtained in 3 to generate the clusters; 5. The clusters generated in step 4 are provided to SMEs to label them with an SDOH category; 6. The SMEs evaluate the clustering and re-categorize any misclassified questions; 7. The categories of the individual SDOH present in our data are compared with the SDOH categories present in the PhenX dataset.

We will now discuss, in more detail, the methods that are utilized to extract the broad categories of data that are acquired from semantically similar, but syntactically distinct, questions in the forms. As this method incorporates the expertise and insight of SMEs, it is sometimes referred to as a “Human-in-the-Loop” approach. Figure 5 is a diagrammatic representation of the flow of data between the various components. The first block of the diagram shows the use of the language model, dimensionality reduction and clustering algorithm to generate clusters. The second block of the diagram shows the “Human-in-the-loop” approach where the clusters are validated by the SMEs and compared with the PhenX data.

#### 3.4.1. Natural language model

We use a pre-trained model all-mpnet-base-v2 (47), a transformer-based natural language model. The model is based on the MPNet architecture and has the highest performance in generating sentence embeddings according to Sentence-Transformers (48). We did not perform any additional fine-tuning on our dataset. The model that was provided by Sentence-Transformers was used in its original form, which has an output dimension of 768. Thus, the output of this model for each question in the SDOH dataset is an embedding that has length of 768.

#### 3.4.2. Dimensionality reduction and clustering

As the embedding produced by the language model has a high dimensionality, we explore dimensionality reduction methods that can be implemented prior to clustering. Therefore, the process we use here is a two-stage approach in which the first stage screens for informative variables (or covariates, or features), while the second stage applies appropriately selected clustering methods on the pre-selected variables. We note here that in the context of model-based clustering of high dimensional data, Bouveyron and Brunet-Saumard (49) indicate that automatic reduction of the dimensionality of the data, without taking into account the goal of clustering, may produce suboptimal results.

**Figure 6:**
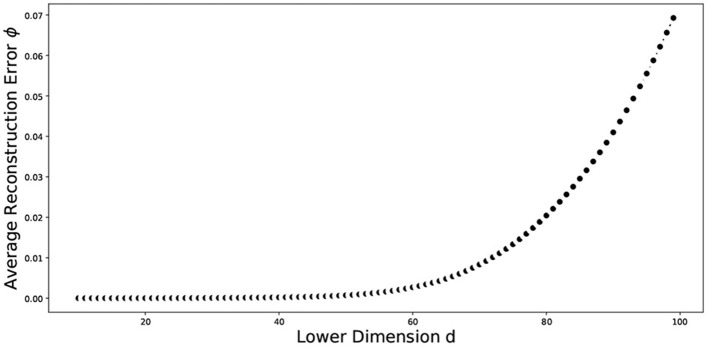
Plot of the average reconstruction error vs dimension of the data. The average was taken over ten random replications of the training data set of size 1,937.

The nonlinear relationships in the data may not be well represented by linear approaches, and therefore linear approaches can perform poorly. Nonlinear dimensionality reduction approaches may be appropriate in this case. We explored different non-linear approaches including manifold learning, kernel PCA (KPCA), isometric mapping (IsoMap), locally linear embedding (LLE), multidimensional scaling (MDS), and uniform manifold approximation and projection (UMAP) on the word embeddings. We implemented clustering techniques from three different categories, which are as follows: partitional methods, spectral methods and hierarchical methods. Partitional methods, such as the k-means and spherical k-means, decompose a data set into a set of disjoint clusters. Spectral methods use a similarity matrix to partition points into disjoint clusters. Hierarchical clustering methods, such as the bisecting k-means, complete linkage, Ward linkage and BIRCH generally build a hierarchy of clusters either by the top-down or bottom-up approach. Some pertinent instances of application of these methods for text clustering include: k-means in Costa and Ortale (50), spectral clustering in Schindler et al. (51), bisecting k-means in Abuaiadah (52), complete Linkage in Abd Rahman et al. (53), Ward linkage in Shehata (54), and BIRCH in Gupta and Rajavat (55). With the exception of UMAP and spherical k-means, all the dimensionality reduction and clustering algorithms mentioned above have been implemented in Python using the Scikit-learn library (56). UMAP was implemented using its own library (57). The locally linear embedding algorithm was proposed in Roweis and Saul (58). In our implementation, we used *K* = 5 neighbors and calculated the reconstruction errors for several lower dimensional representations ranging from *d* = 2 to *d* = 100. We plot the average reconstruction error Φ(*Y*) versus the number of components (*d*) to determine the best number of components for us. Table 1 presents the summary statistics associated with the average reconstruction error, and Figure 6 plots the average reconstruction error, the average taken over 10 random replications of the clustering process using a data set of size 1,937. Spherical k-means is a variant of the normal k-means technique, which is widely used for data clustering. The primary distinction between regular k-means and spherical k-means is that the latter represents data points and cluster centroids as points on a unit sphere. This makes it possible to compute the distance between data points and cluster centroids more efficiently. The spherical k-means works in the same way as the standard k-means algorithm, with the key difference being the distance measure. The spherical k-means employs the cosine distance (also known as cosine dissimilarity) as the distance measure, and it is commonly used in document clustering and other applications with high-dimensional vectors. In our research, we employed an implementation of spherical k-means as proposed in a study by Kim et al. (59). The study introduced a technique for fast initialization of cluster centroids, reducing the computational cost of the algorithm. Additionally, the study proposed a method for projecting sparse centroids, which uses a sparse representation of the centroids to decrease the computational expenses of the algorithm. This sparse representation can significantly decrease the number of non-zero entries in the centroids, thereby reducing the computational cost of the algorithm. The implementation can be found in (60). The parameters used were: max_iter = 10, init = similar_cut, sparsity=minimum_df, minimum_df_factor = 0.05. The “minimum_df_factor” parameter is used to specify the minimum number of documents in which a term must appear as a proportion of the total number of documents. This parameter is used to filter out rare terms that may not be informative for clustering. For example, if minimum_df_factor is set to 0.05, then terms that appear in fewer than 5% of the documents will be removed, helping the reduction of dimensionality of the data and speeding up the clustering process. It also helps to increase the interpretability of the clusters by reducing the number of irrelevant terms.

**Table 1:** The table indicates the reconstruction error values as a function of the dimension in the neighborhood of the chosen optimal dimension.

**Dimension (d)**	**Average reconstruction error**
20	8.35 × 10^−6^
30	6.47 × 10^−5^
35	1.22 × 10^−4^
40	2.14 × 10^−4^
45	3.85 × 10^−4^

The average was taken over ten random replications of the training data set of size 1,937.

**Figure 7:**
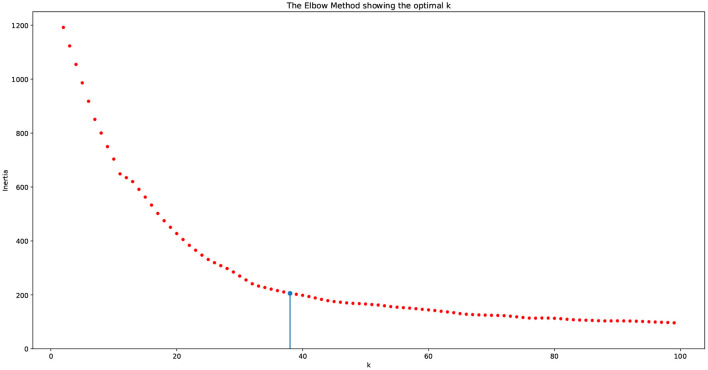
Elbow plot for choosing the number of clusters. The vertical line indicates the number of clusters produced by the algorithm, which equals 38.

The above described process entails the selection of a pair of dimension reduction method and clustering algorithm for identifying the number of components to be kept and subsequently used for identification of the number of clusters. Reproducibility of both, the process followed and the findings, is important. To assess the performance of the different methods used and decide on the number of clusters, we used a variety of internal validation metrics, such as Calinski-Harabasz (CH) index, silhouette coefficient, and the elbow plot to identify the pair of clustering algorithm and dimensionality reduction methods that are appropriate for our data. The combination of LLE and spherical k-means performs best. The final dimensions used is equal to 35 (Table 2). The total number of clusters provided by LLE and spherical k-means from the elbow plot is 38. Figure 7 depicts the elbow plot we used to identify the total number of clusters.

**Table 2:** Summary statistics of the reconstruction error when lower dimension *d* = 35 over ten random replications of the training data set of size 1,937.

**Minimum reconstruction error**	**6.36 × 10^−5^**
Maximum reconstruction error	1.92 × 10^−4^
Median reconstruction error	1.10 × 10^−4^
IQR of reconstruction error	9.03 × 10^−5^
Mean of reconstruction error	1.22 × 10^−4^
Standard deviation of reconstruction error	5.14 × 10^−5^

As seen in the elbow plot, we determined that 38 is the ideal number of clusters produced by using the language model, dimensionality reduction, and clustering algorithm.

**Table 4** presents the definitions of the labels of the final clustering using the collaborative approach of automation and labeling by the SMEs.

#### 3.4.3. Evaluation of the cluster model

The procedure described in the previous section produces a clustering model, in which each cluster contains syntactically different questions corresponding to the same SDOH variable. In this section, we describe an evaluation procedure that relies on the use of a cluster quality measure, called the in group proportion. We then measure the agreement of clustering against the PhenX data set.

#### 3.4.4. Computing IGP

Methods for assessing the reproducibility of clustering patterns available in the literature include bootstrap and testing procedures for the significance of clustering. The main idea in computing the IGP index can be described as follows. First, we have two independent sets of data, where one set is called the training set and the second the test set. These two sets are not required to have the same size. In the next step, we cluster the training and test data into k clusters. Finally, we measure how well the training set cluster centers predict co-membership in the test set. For each pair of test observations assigned to the same test cluster, we determine whether they are also assigned to the same cluster based on the training centers.

The total size of our data set is 3,582 questions. We randomly partition this set into two subsets, a training set with size 1,937 and a test set with size 1,645. These sets are independent of each other by construction. We developed our clustering model using the training set and compute IGP using the R package “clusterRepro” (Version 0.9, October 12, 2022).

Additionally, we tested our methods over 10 independent runs to further evaluate the reliability of the results. Table 3 presents the summary statistics of the IGP over the 10 runs. Notice that all means of the IGP scores are fairly high, indicating the validity of the different clusters.

**Table 3:** Summary statistics of the IGP scores over 10 runs.

Iteration	Median	IQR	Mean	SD	Q1	Q3	Range
0	0.91	0.118	0.896	0.077	0.837	0.955	0.267
1	0.915	0.115	0.853	0.191	0.841	0.955	1.0
2	0.924	0.086	0.897	0.088	0.87	0.956	0.385
3	0.918	0.164	0.879	0.129	0.815	0.979	0.5
4	0.93	0.122	0.891	0.116	0.842	0.964	0.625
5	0.948	0.086	0.922	0.095	0.898	0.984	0.5
6	0.927	0.088	0.879	0.174	0.873	0.961	1.0
7	0.935	0.085	0.914	0.085	0.885	0.97	0.333
8	0.919	0.14	0.891	0.098	0.825	0.965	0.333
9	0.922	0.135	0.886	0.116	0.83	0.965	0.6

#### 3.4.5. Comparing with the PhenX dataset

**Figure 8:**
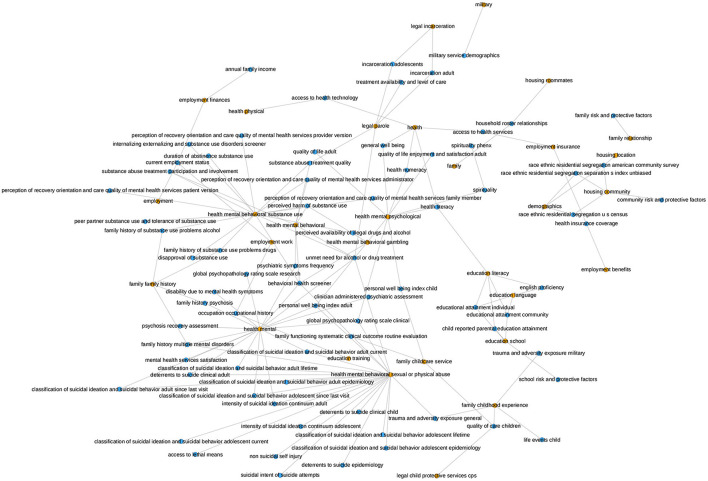
This graph illustrates the mapping of the PhenX SDOH Toolkit Protocol Names to the SDOH categories in our data. The orange nodes represent the SDOH categories in our data and the blue nodes represent the PhenX categories. The mapping was generated by comparing the cosine similarity between the names of the Protocols and the names of the SDOH categories in our dataset. The presence of an edge between a blue node and an orange node signifies that the PhenX category and the SDOH category in our data were found to be semantically similar according to the cosine similarity. The occurrence of many connections from one SDOH category in our dataset to several PhenX data categories indicates that our category is determined to be comparable to more than one category in the PhenX data.

**Figure 9:**
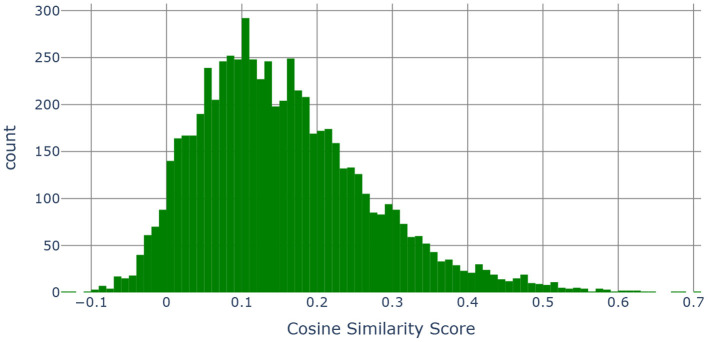
Histogram of the cosine similarities between the embedding vectors of “category, subcategory 1, subcategory 2, subcategory 3 and the definition” and “PhenX categories”.

The PhenX Toolkit (consensus measures for **Phen**otypes and e**X**posures) provides recommended standard data collection protocols for conducting biomedical research. The protocols are selected by Working Groups of domain experts using a consensus process, which includes the scientific community (61). In 2018, the National Institute on Minority Health and Health Disparities (NIMHD) funded an administrative supplement to the PhenX project to select high-quality standard measures related to SDOH for inclusion in the PhenX Toolkit (62). We match the SDOH categories for which we have data with the measures available in the PhenX toolkit. Figure 8 presents the mapping of the PhenX SDOH toolkit protocol names to the SDOH categories identified in our data, while Figure 9 presents a histogram of the cosine similarities between the PhenX categories and our embedding vectors.

The main idea is to combine the category, subcategory 1, subcategory 2, subcategory 3 and the definition (see Table 4) together into a single string and compare those with the names of the measures contained in the SDOH PhenX toolkit. Using the MPNet language model, we generated 768 dimensional embedding vectors for each of the measure names in the PhenX Toolkit as well as for the category, subcategory 1, subcategory 2, and subcategory 3 and the definition combined. We then computed pairwise cosine similarities (63) to measure the similarity between two vectors in an inner product space. Cosine similarity is widely used in text analysis. Mathematically, if *x* and *y* are two *d* dimensional vectors, then - $sim\left(x,y\right)=cos\theta =\frac{x\cdot y}{\left||x||||y|\right|}$ where ||*x*|| is the euclidean norm of vector *x* = (*x*_1_, *x*_2_, ..., *x*_*d*_) defined as $\sqrt{x_{1}^{2}+x_{2}^{2}+...+x_{d}^{2}}$. The cosine similarity always belongs to the interval [−1, 1].

**Table 4:** Final clustering table.

**Category**	**Subcategory 1**	**Subcategory 2**	**Subcategory 3**	**Definition**
Health				General category, anything “medical” or affecting the physiologic functioning of the body (i.e., physical) or mind (i.e., mental).
Health	Physical			Physiological (e.g. HIV testing), neurological (e.g., sleep)
Health	Mental			Psychiatry (emotion, mood, hallucination, nightmare, eating disorder, contemplating suicide/homicide, developmental/learning disabilities)
Health	Mental	Psychological (Non-medical)		Confidence or ability to complete the activities of daily living, stressors, attitude, meditation, understanding, comprehension, awareness, judgement, insight, object recall, self-help, life goals, life plan, sexual orientation (i.e., gender)
Health	Mental	Behavioral		Addiction, losing control, behavioral therapy (group/individual)
Health	Mental	Behavioral	Substance Use	Role of substance use, treatment, detox, relapse
Health	Mental	Behavioral	Gambling	Addicted to playing games of chance for money
Health	Mental	Behavioral	Sexual or Physical Abuse	Violence to others/self, general violence/domestic violence, action/plan for homicide or suicide.
Family				Group of adults and their children living together or with shared experiences
Family	Family History			Family history of any illnesses, conditions, family of origin
Family	Relationship			Support system, sex (condom), relationship with children or living with children
Family	Childhood Experience			Experiences growing up, trauma, fear, foster care, upbring
Family	Childcare Service			Childcare needs
Education				Process of giving or receiving systematic instruction.
Education	Training			Vocational
Education	Language			Secondary Language
Education	School			Learning style, educational plan, grade, education problems, educational goals, degree
Education	Literacy			Reading, writing, arithmetic
Employment				Condition of having paid work
Employment	Work			Workplace, work environment
Employment	Finances			Income, salary
Employment	Insurance			Medicare, medicaid, private insurance
Employment	Benefits			Social security, disability, assistance (employment assistance program [eap], social assistance, etc)
Housing				Shelter or living quarters
Housing	Location			Physical space (i.e., building, structure, homelessness)
Housing	Roommates			Household activities, number of people who live in households
Housing	Community			Neighborhood, county, transportation
Leisure				Free time, hobbies, interests, activities
Legal				Related to or the process of the law
Legal	Legal History			Court, prior conviction, prior arrest, sentencing
Legal	Parole			Mandated treatment
Legal	Incarceration			Jail, Prison
Legal	Child Protective Services (CPS)			Child protection against mistreatment
Demographics				Age, sex, race, ethnicity, primary language, zip code
Military				Relating to or the characteristic of the armed forces
Spirituality				Quality of being concerned with human spirit or soul.

The definitions of the 36 clusters presented below represent the fusion of all reports and are obtained via automation and validation by the SMEs. The gray boxes are left intentionally blank.

#### 3.4.6. Determination of threshold

**Figure 10:**
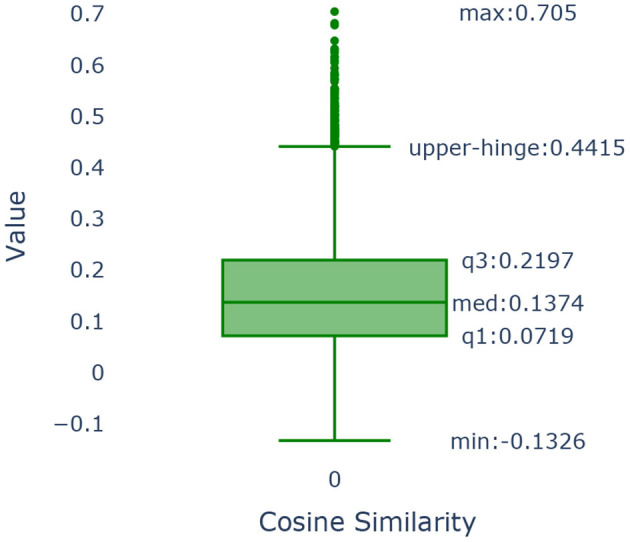
Boxplot of the cosine similarities between the embedding vectors of “category, subcategory 1, subcategory 2, subcategory 3 and the definition” and “PhenX categories”.

To determine the threshold, we use a data-driven method that is based on the use of the boxplot of the cosine values as shown in Figure 10.

The boxplot is a graphical method that demonstrates key characteristics of the distribution of the cosine similarities among the PhenX categories of the SDOH and those found in our data. We use as our cut-off value the upper hinge of the boxplot. The upper hinge is defined as the third quartile of the data plus 1.5 × *Interquartile Range*. Values of the cosine similarity that are greater than the upper hinge imply that the pairs of text to which they correspond are similar. The upper hinge of a boxplot corresponds to indicating the point that is approximately 3 standard deviations away from the mean should the sample follow a normal distribution. In our case, the value of the upper hinge is 0.4415. Our text consisted of the category, subcategory 1, subcategory 2, subcategory 3 & the definition as illustrated in Table 4. Our text was compared to the measure name in the PhenX data set and were found to be similar. The cosine similarity values show that almost 86.11% of the categories in our data set map to one or more categories.

## 4. Data quality and the pursuit of reproducibility

The incremental nature of data exploration is at odds with the needs of reproducibility. The former is *ad-hoc* and exploratory, while the latter requires deliberate, methodical documentation of process, including the reasoning behind specific choices. As already discussed, a significant portion of our data preparation and analytical work relied on a computational notebook called Vizier (36, 39, 64, 65). We now discuss the design of Vizier, and how it works to make it easier to track the processes that resulted in visualizations, models, and other research artifacts.

Computational notebooks like Jupyter (66), Apache Zeppelin (67), or Vizier provide users with a close analog of a scientific notebook that tracks the evolution of their scientific process. As users of a computational notebook append units of code (called ‘cells') to the notebook, the code is run and its results are shown inline. Code cells can be supplemented by documentation cells that exist purely for the user to record their thoughts. In principle, the notebook records the full set of steps required to reconstruct a scientific artifact.

In practice, there exist several challenges in maintaining and using this record. First, many computational notebooks allow non-linear edits to the notebook: a user may return to and revise earlier steps in the notebook if they realize they made a mistake. The final revision of the notebook may not adequately describe the context in which a particular piece of code was written, making it difficult to understand why a particular choice was made. Second, as a notebook becomes increasingly complex, it becomes difficult to follow the logic behind how a particular artifact was constructed. Similarly, even if the process of an artifact's construction is well documented, it can be difficult to keep track of which documentation is relevant to that artifact in a complex notebook.

### 4.1. How do we ensure the reproducibility of our work?

Effective reproducibility requires a record not only of what the user did and when, but also why he/she did it. It is not realistic to expect software to understand the user's reasoning in general.

#### 4.1.1. Automating context tracking

**Figure 11:**
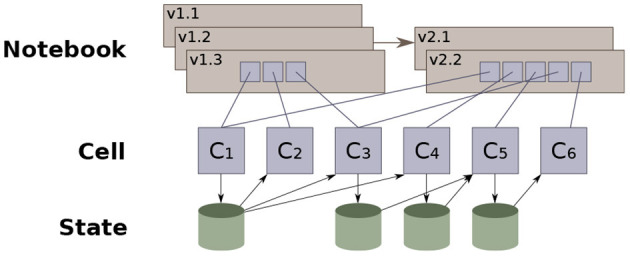
Vizier's notebook versioning data model (36).

Instead, Vizier records as much as possible of the context in which a decision was made; making it easier to infer reasoning in retrospect. Concretely, each modification to the notebook is recorded by Vizier as a notebook revision, along with metadata about what changed in the notebook, and what remained unchanged. Figure 11 illustrates a simplified version of the model: each edit to the notebook generates a new revision, and users may manually elect to backtrack and “branch” an older version of the notebook. Each revision is a sequence of references to cell descriptions that provide the code or documentation that defines the cell. Cell descriptions may be shared across multiple revisions, both minimizing wasted space, as well as providing an easy way to compute the differences between two workflows.

Revisions also track the results of running each cell on the output of the prior cell—We call this the “state” of the notebook at the cell.

### 4.2. State and provenance

Vizier views state as a collection of name-value pairs (i.e., variables and the corresponding values). We refer to values as the notebook's ‘artifacts,' and these may include data sets, models, data visualizations, or indeed even simple variable values that are passed from one cell to the next. State evolves along two dimensions: notebook order and revision order. Each code cell interacts with the state; the cell's code reads from the state generated by prior cells, and generates changes to the state that are visible to subsequent cells. Vizier checkpoints the state after each cell finishes running. We refer to this sequence of cells as the state's evolution in notebook order. As the notebook is revised, non-linear updates modify portions of the state, which are likewise checkpointed after each cell is run. We refer to the sequence of states resulting from edits to the notebook (non-linear or otherwise) as revision order.

Checkpointing in both program and revision order makes it possible to quickly reconstruct the full context in which a user decided to edit a cell, as well as the differences before and after the cell was run. In particular, Vizier records which artifacts a given cell interacted with in a given revision of the notebook. This information, in aggregate across the entire notebook, defines a set of dependencies for each artifact produced by the notebook, and is referred to as the *provenance* of the artifact.^2^

**Figure 12:**
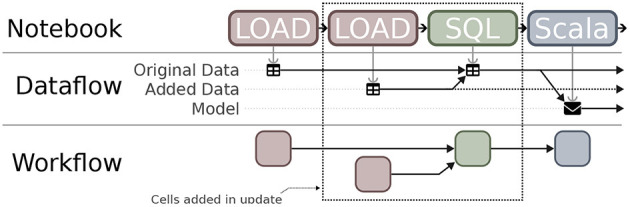
Extracting dataflow and workflow provenance from notebooks.

The notebook's provenance—the artifacts each cell reads and writes—defines a dataflow graph that shows how each specific artifact was derived. For example, consider a workflow where a data set is loaded and used to derive a model. A simplified version of the dataflow graph that Vizier generates for this workflow is shown in Figure 12, excluding the cells in the dotted box. The figure shows that the ‘Model' artifact was derived from a single data set (‘Original Data'). If additional data are discovered, they can be easily integrated into the workflow: The data scientist adds two new cells, one to load and clean the additional data, and one to merge the two data sets together (e.g., using SQL). The dataflow diagram is updated, showing the ‘Model' artifact derived from the output of the merge cell, which itself was derived from the two source datasets.

#### 4.2.1. Ensuring correctness

Non-linear edits to notebooks come with another challenge: staleness (68). When a non-linear edit is made to one cell, the changes may affect some cells that follow it. A common criticism of many computational notebooks (69, 70) is such edits lead to stale cells. These are cells that appear normally in the notebook, but that read from state that no longer exists, and as a result will fail (or worse, produce different outputs) when the notebook is restarted. When performing a non-linear edit, users are expected to identify stale cells manually and re-run them (labor-intensive), or to periodically re-evaluate the entire notebook (slow).

From the notebook's dataflow graph, Vizier derives a workflow, or dependency graph that captures inter-cell dependencies. Recall the example from Figure 12 where the data scientist adds two new cells to load and merge new data into the original workflow. The dataflow graph changes, linking the input of the model-building cell to the output of the (new) cell that merges the data sets. Vizier recognizes that the model-building cell needs to be re-evaluated to keep the output fresh, but that the original data loading (and cleaning) cell's output can be safely re-used.

Concretely, Vizier encourages users to keep notebooks up-to-date by automatically identifying and re-evaluating stale cells. This ensures that (i) users are immediately notified if non-linear changes break a portion of their notebook, and (ii) users later viewing the output of those cells are guaranteed not to be viewing stale outputs.^3^

A key challenge is when the notebook requires users to take actions outside of the notebook. For example, a common pattern is for one portion of a notebook to generate an excel spreadsheet, which the user edits before running the second portion of the notebook. Vizier addresses this use case, and others, by providing a spreadsheet-style data editor that tracks the user's actions as they edit a data set. Crucially, a record of the user's actions (71) is stored in the notebook and may be replayed if the source data change (36).

### 4.3. How do we help users to track down bugs?

Extensive context (i.e., provenance) tracking is useful, but simply displaying all collected information also includes an overwhelming amount of data not relevant to the user's immediate task. Instead, Vizier leverages the collected provenance information to support several filtered displays, each designed to help users answer specific questions about data and artifacts.

#### 4.3.1. Dependency tracking

Common questions asked by data scientists about their data are “where did a data set (or model, visualization, etc, come from?” or “how is a data set used?” For example, a user may wish to know which cells were involved in the artifact's creation as part of a sanity check, or which models were affected by a training data set that was since identified as flawed.

Fundamentally, both of these questions ask about the dependencies of a given artifact. Vizier maintains sufficient state to provide several tiers of user interfaces, from lightweight but less informative to heavier-weight solutions that are more likely to address the user's question. The lightest-weight approach relies on a portion of Vizier's user interface called the “Table of Contents,” which summarizes every cell and artifact in the notebook. Hovering over a cell in the notebook highlights (i) the direct dependencies of the cell (i.e., upstream cells that generated artifacts that the hovered cell reads from), (ii) the cell's transitive dependencies (i.e., the cells that these cells read from), (iii) cells that depend directly on the hovered cell's outputs, or (iv) cells that depend transitively on the hovered cell. Similarly, hovering over an artifact highlights dependencies with respect to the artifact.

Hovering is meant to be lightweight and quick, but particularly when the table of contents is large, it may be difficult for the user to see all of the dependencies. As a second tier, Vizier allows users to filter the notebook itself by dependencies. This acts like highlighting, but provides a read-only view of the notebook that shows only cells that contribute to (respectively, rely on) the inputs (resp., outputs) of the indicated cell, or on the indicated artifact. Finally, Vizier can provide a visual representation: Figure 12 shows, visually, the dependencies between the notebook's cells and their artifacts.

#### 4.3.2. Fine-grained data dictionaries

Where possible, Vizier tracks the so-called “fine-grained” provenance of its artifacts; retaining a record of the precise logic used to derive one artifact from another. For example, when a database query is used to derive a data set by joining together two other data sets, Vizier retains the query. From this information, it is possible to infer relationships, not only between artifacts, but between their components. For example, fine-grained provenance can be used to infer which records in the source data sets were used to derive a record in the output data set.

Vizier makes use of fine-grained provenance for data documentation. Data sets are commonly documented through “data dictionaries” that outline, often in exacting details, the nuances and unique features of the data set. This information is helpful, but can be overwhelming, particularly in the early stages of data exploration. Vizier allows users to define more targeted forms of documentation through a feature of Vizier called Caveats (40, 64, 72, 73). These annotations are propagated through the notebook using fine-grained provenance.

Vizier identifies portions of a data set (e.g., cells, rows, columns) that have been annotated by a provenance value, drawing the user's attention to the fact that there may be relevant documentation available. The user can then retrieve the documentation that applies to the portion of the data set that they are interested in (e.g., by clicking on a button next to a highlighted cell); only relevant documentation will be displayed, allowing them to focus their attention where it is needed.

### 4.4. The shape watcher

One of the specialized cell types that Vizier provides is called the shape watcher, which records a set of data set features called ‘facets': (i) The set of attributes of the data set, (ii) The type and nullability of each attribute, (iii) The range of values for an ordinal attribute, and (iv) The set of distinct values for a categorical attribute. When a shape watcher lens is initialized, it detects facets relevant to the data set. Subsequent updates to the data set at that point in the notebook, for example as a result of newly added data, trigger the shape watcher. The shape watcher flags any facets that the new data set violates.

For example, consider one data source that initially uses the symbols ‘M' and ‘F' to indicate sex, but where the data dictionary changes, and new records switch to using the terms ‘Male' and ‘Female'. The shape watcher would: (i) Warn the user that the data set now includes a set of records that where the ‘sex' attribute has an unexpected categorical value, and (ii) Flag all of the new records with Caveats so that all artifacts derived from the data set are marked with warnings about the error.

## 5. Discussion

### 5.1. Real world importance of SDOH data

In this manuscript, we have described a pipeline to enable collection, integration, and effective use of SDOH data derived from an underserved population. Our study population is derived from individuals with OUD who participated in a randomized controlled trial assessing the effectiveness of telemedicine with onsite DAA administration compared to offsite referral for HCV treatment. Analyzing SDOH data requires understanding of which determinants are important to measure. It also requires data collection from non-traditional and non-health data sources (74).

The importance of SDOH data is increasingly recognized. Segregated communities in the US have been major drivers of healthcare disparities, and this history emanates from redlining. Redlining was a practice whereby lending institutions restricted mortgages to African American applicants in certain neighborhoods, which led to their concentration in often less desirable neighborhoods. One goal of the renewed focus on SDOH is to ensure health equity, which requires collection of SDOH and community-level data including location of residence, zip code, quality of food availability, and ethnic/racial neighborhood composition. While the COVID-19 pandemic underscored the importance of comorbidity data collection, other important data elements are evaluation of structural racism, under or lack of insurance, poor quality of care, and food and housing insecurity. An important consideration in the collection of these data is society's stigmatization of people with OUD. People with OUD typically interpret society's views of addiction as a moral failing (75–77). Healthcare providers, especially those unfamiliar with the treatment of addiction, have historically perceived people with OUD as irresponsible and nonadherent to medical care (78, 79). Thus, truthfulness of responses to questions ascertaining SDOH information appears to depend on the trust and comfort between the people with OUD and the individuals attempting to collect the information. In the collection of SDOH data, the ACP recommends that data must be granular and inclusive of all personal identities to more accurately identify socioeconomic trends and patterns (1). In a recent review, for example, Taylor et.al. found that interventions targeted to address SDOH have a positive outcome on health and healthcare spending and that new workflows are needed to administer SDOH assessments, especially as the US healthcare system transitions to value-based care (22).

Addressing underlying factors that impact health and wellness is a cost-effective means to prevent chronic diseases and health inequities and improve overall population health. While preventative medicine is less expensive than treatment, the same applies for social factors. It is estimated that 70% of health is determined by social factors and only 20% is determined by clinical care (80, 81). Studies have found associations between unemployment, homelessness, drug use, and poor mental health in diverse communities. Family relationships and support are also important to consider as adolescents and young adults are likely to be influenced by behaviors they observe or perceive as acceptable based on childhood experiences (82). Interestingly, it has been proposed that internet access, dependent on place of residence, is another important SDOH to consider (81). Another consideration for accurate SDOH data collection is participant health as well as cultural and educational literacy. People with OUD have been shown to have low to moderate health literacy levels (83–85), and health literacy is an extremely important predictor of health status (86). Another factor, racism, has been significantly related to poor overall health, especially mental health, as the association between racism and poor mental health was twice as large as the association between racism and poor physical health. (87).

#### 5.1.1. Data aggregation issues

Two main issues concern data aggregation, bad data acquisition and bad data management. To reliably, accurately, and confidently acquire SDOH data in clinical environments, trust needs to be engendered at the patient, health system, and governmental (i.e., local, state, federal) levels, each with their own potential concerns that must be addressed. In terms of patients, particularly those from underserved populations, they need to have confidence that their health information will remain secure and confidential. Collecting sensitive data in venues that patients describe as safe spaces by people who are familiar with their situations can facilitate patients' trust in the process of data acquisition, transmission, and usage. Since the OTP is described by people with OUD as a “safe space" (18, 19), they are more likely to trust the clinical and non-clinical staff in a non-judgmental, destigmatizing environment compared to conventional healthcare settings, such as the emergency department, urgent care, or primary care (29). In our context, data were acquired by OTP staff and healthcare providers, which has been shown by others to largely circumvent stigma encountered outside of the OTP (13, 14). Therefore, people with OUD are willing to provide truthful answers, enabling more accurate SDOH data collection, when they trust the staff and feel respected.

The study was conducted at 12 sites across NYS, all overseen by the same state agency (OASAS). We were able to obtain permission from OASAS to utilize data collected for clinical purposes to extract relevant SDOH. Over the course of the study, the research team actively participated in OTP activities and workflows, demonstrating trust, respect, and familiarity from an external entity. The research team introduced their IT specialist (MB) to the staff of each OTP involved in SDOH data collection. This transfer of trust permitted the IT specialist to work with the OTP staff to collect SDOH data. Data collection challenges, however, varied by site. For example, one OTP had to enter the information from the intake forms into a spreadsheet to share with the IT specialist due to difficulty obtaining archival clinical information. Other sites had to obtain intake forms from their archives and mail paper copies to the research team for data entry. Other sites had difficulty downloading SDOH forms from OASAS, so the IT specialist had to train OTP staff and develop software to download and only retain data relevant to study participants. All data entry of SDOH forms were reviewed by different members of the research team for accuracy. Data entry was documented in a tracking spreadsheet with dates of conduct, dates of entry, and confirmation codes. The tracking spreadsheet was used extensively for cross-referencing input and output data, as well as correcting computational and human data entry errors.

Beyond the issue of what types and how data should be collected, there are issues of how the data are to be handled once they are collected. The specific tasks to be considered include data aggregation, secure transfer, and merging with already existing data sets. In our context, data collected have been syntactically different but semantically very similar, making integration feasible. As the scope of the project expands to other healthcare settings, we expect a greater diversity of attributes to appear, including the possibility that additional attributes may become available for existing records. Thus, even in this controlled setting, defining a single unified data model is impractical. We need a data model that will allow us to transfer this mass of heterogeneous data into a clinical setting. It is crucial that this model must be extensible, allowing new data to be easily linked to and integrated into existing data. The integration process should adapt and evolve, with each integration effort making it easier to integrate new data. The process should also be aware of the uncertainty that it induces and able to communicate this uncertainty to users of the integrated data (e.g., through provenance). For example, subtle phrasing differences across two data collection instruments may render them incomparable with respect to a specific study. Finally, for such a process to be practical, it must be commoditized or packaged in a comprehensive tool. Vizier's workflow system is a first step in this direction, but it remains an open challenge for the data management community how to structure such a tool.

#### 5.1.2. Maintenance of reproducibility

How can we assess the reproducibility of the identified clusters that contain questions associated with the different SDOH? In our case, we first assessed the validity of the identified clusters by computing the IGP scores. Further, we were able to compare the SDOH categories identifed in our data with those present in the PhenX data. Our procedures used Vizier, a computational notebook. We ensured reproducibility by using Vizier to record changes to the data, when they occurred, and why, as each addition to the notebook makes an edit and a new version of the data. Another Vizier function is producing a workflow, or dependency graph, that captures intercell dependency. As data modeling and analysis progresses, new output can be merged into the datasets.

### 5.2. Research and policy implications

With the growing importance of SDOH in many dimensions, as described throughout this article, it is incumbent on the research community to develop reliable, validated approaches to utilize the data in a straightforward manner with reproducible results. While the topic of policy issues related to SDOH and relevant data acquisition is quite broad, due to space constraints, we will limit our comments to address data collection of underserved populations to inform inclusivity and comprehensiveness of healthcare systems. Without complete data, stakeholders, including policymakers, physicians and other health professionals will be unable to make highly informed, evidence-based decisions regarding care to communities most impacted by SDOH. Several relevant recommendations have recently been put forward by the ACP (88).

1. Data sharing-Data collected on testing, infection, hospitalization, and mortality during a pandemic or in response to screening and surveillance for infectious diseases (i.e., HCV or HIV) should be shared with all relevant stakeholders including government agencies at all levels, academic researchers, and policymakers responsible for analysis of healthcare utilization trends and forecasting for future growth.

2. Health literacy and culturally relevant data acquisition tools should be available to assist in the collection of self-reported data. Similarly, resources should be made available to clinicians so that they are able to implement health literacy interventions and to satisfactorily address cultural, informational, and linguistic needs of their patients.

3. With regard to underserved populations, if we desire a more inclusive healthcare system, then prioritization of data collection among certain underserved populations may be necessary. Especially in reference to pregnant women, the ACP has supported establishing maternal mortality review committees (MMRCs) that would be charged with collecting relevant data, identifying causes of maternal death, and developing strategies to prevent pregnancy-related death and improve maternal outcomes. In the 38 states where MMRCs have been established, they have reduced maternal mortality by 20– 50% (89), although 12 states have not established MMRCs (90).

Why is data prioritization needed? Timely access to accurate and comprehensive data is crucial to addressing SDOH. In many areas of SDOH, there has been a recent transition to electronic reporting. Perhaps the lessons learned as explained in this article can assist in the utilization of these data.

Reproducibilty is a foundational concept to scientific and technical research because it allows confirmation of the validity of the reported findings. Well documented data acquisition workflows facilitate data reproducibility and optimal research practices. Reproducible workflows, in turn, facilitate reproducible analyses and the identification of potential errors in the data and the analysis. Clustering methods provide a powerful tool that is versatile in its use. Detecting and determining the presence of clusters and obtaining reproducible results for the clustering procedures has been an important concern. Kapp and Tibshirani (35) propose the nonparametric IGP method for evaluating cluster reproducibility. Techniques for testing the significance of the clustering results have also been proposed in the statistical literature (91). We tested the reproducibility of our clustering by comparing our clustering with the one provided by the PhenX data set. More work is needed in this area to derive methods that can be used with many data structures and dimensions of the data.

Data curation and computational analysis play a major role in modern scientific endeavors. Ensuring reproducibility of these procedures requires tracking not only its individual steps (i.e., what choices were made), but the context in which those steps were taken (i.e., why the choices were made). To support reproducibility, tools for data curation and analysis need to collect both forms of metadata. More than this, reproducible data science technology should give its users the tools they need to understand the metadata — tracking the relationship between constructed artifacts as well as viewing the context in which a particular item was created. We have observed, in particular, a need for context-aware documentation [e.g.,as in Kumari et al. (64)], not only to provide context for data, but also as a sort of “guard rail” for data science. Crucially, such guard rails can not be one-size-fits-all; even within a single domain, minor changes in context (e.g., the addition of additional attributes) can invalidate one form of analysis, while making another valid. Rather, an ideal tool would build and track institutional knowledge, developed through experience in a domain and working with specific categories of data.

This paper addresses a fundamental issue in the expanding role of SDOH as interventions targeted to improve healthcare equity and disparities continue to evolve. We have outlined a process to obtain high-quality, reproducible data from clinical records collected longitudinally. We have outlined a process for data extraction, acquisition, and preparation for analysis using a computational notebook approach that has incorporated several features to enhance reproducibility. The system readily incorporates external data sources for analysis as well as for comparison and as a benchmark. Given the growing importance of SDOH, the procedures outlined here may be highly transferable to other settings and populations.

## Data availability statement

The original contributions presented in the study are included in the article/supplementary material, further inquiries can be directed to the corresponding author.

## Ethics statement

The studies involving human participants were reviewed and approved by University at Buffalo Institutional Review Board. The patients/participants provided their written informed consent to participate in this study.

## Author contributions

MM: conceptualization, methodology, validation, formal analysis, investigation, resources, writing—original draft, writing—review and editing, supervision, project administration, and funding acquisition. OK: methodology, software, validation, formal analysis, investigation, resources, writing—original draft, writing—review and editing, supervision, and project administration. MB: methodology, software, validation, formal analysis, investigation, resources, writing—original draft, writing—review and editing, and visualization. RM: methodology, software, validation, formal analysis, investigation, resources, data curation, writing—original draft, writing—review and editing, and visualization. AD: investigation, resources, data curation, writing—review and editing, and project administration. AT: conceptualization, methodology, investigation, resources, data curation, writing—original draft, writing—review and editing, supervision, project administration, and funding acquisition. All authors contributed to the article and approved the submitted version.

## Footnotes

^1^ https://vizierdb.info

^2^The terms Lineage or Pedegree are also used in some communities.

^3^Vizier has a “frozen cell” mode that allows users to indicate that retaining a stale output is desirable; cells with this feature enabled are prominently identified.

## References

[1] Daniel H Bornstein SS Kane GC. Addressing social determinants to improve patient care and promote health equity: an american college of physicians position paper. Ann Internal Med. (2018) 168:577–8. 10.7326/M17-2441 29677265

[2] Galea S Tracy M Hoggatt KJ DiMaggio C Karpati A. Estimated deaths attributable to social factors in the United States. Am J Public Health. (2011) 101:1456–65. 10.2105/AJPH.2010.300086 21680937 PMC3134519

[3] Chetty R Stepner M Abraham S Lin S Scuderi B Turner N. The association between income and life expectancy in the United States, 2001-2014. JAMA. (2016) 315:1750–66. 10.1001/jama.2016.4226 27063997 PMC4866586

[4] Crowley R Kirschner N Dunn AS Bornstein SS. Health and public policy to facilitate effective prevention and treatment of substance use disorders involving illicit and prescription drugs: an american college of physicians position paper. Ann Internal Med. (2017) 166:733–6. 10.7326/M16-2953 28346947

[5] National Drug Threat Assessment (2011). Available online at: http://www.justice.gov/archive/ndic/pubs44/44849/44849p.pdf (accessed on October 18, 2022).

[6] New York State Office of Addiction Services Supports. Person-Centered Care Guidance (2018). Available online at: http://oasas.ny.gov/system/files/documents/2020/01/oasasperson-centeredcareguidance.pdf (accessed on October 20, 2022).

[7] Hepatitis C. (2022). Available online at: http://www.who.int/news-room/fact-sheets/detail/hepatitis-c (accessed on October 21, 2022).

[8] Amon JJ Garfein RS Ahdieh-Grant L Armstrong GL Ouellet LJ Latka MH. Prevalence of hepatitis C virus infection among injection drug users in the United States, 1994–2004. Clin Infect Dis. (2008) 46:1852–8. 10.1086/588297 18462109

[9] Edlin BR Carden MR. Injection drug users: the overlooked core of the hepatitis C epidemic. Clin Infect Dis. (2006) 42:673–6. 10.1086/499960 16447113 PMC1611492

[10] Ghany MG Morgan TR Panel AIHCG. Hepatitis C guidance 2019 update: American association for the study of liver diseases-infectious diseases society of America recommendations for testing, managing, and treating hepatitis C virus infection. Hepatology. (2020) 71:686–721. 10.1002/hep.31060 31816111 PMC9710295

[11] US Department of Health and Human Services. Viral Hepatitis National Strategic Plan for the United States: A Roadmap to Elimination (2021-2025). Washington, DC: US Department of Health and Human Services (2020).

[12] Scott N Doyle JS Wilson DP Wade A Howell J Pedrana A. Reaching hepatitis C virus elimination targets requires health system interventions to enhance the care cascade. Int J Drug Policy. (2017) 47:107–16. 10.1016/j.drugpo.2017.07.006 28797497

[13] Biancarelli DL Biello KB Childs E Drainoni M Salhaney P Edeza A. Strategies used by people who inject drugs to avoid stigma in healthcare settings. Drug Alcohol Depend. (2019) 198:80–6. 10.1016/j.drugalcdep.2019.01.037 30884432 PMC6521691

[14] Muncan B Walters SM Ezell J Ompad DC. “They look at us like junkies”: influences of drug use stigma on the healthcare engagement of people who inject drugs in New York City. Harm Reduct J. (2020) 17:53. 10.1186/s12954-020-00399-8 32736624 PMC7393740

[15] Eveleigh RM Muskens E van Ravesteijn H van Dijk I van Rijswijk E Lucassen P. An overview of 19 instruments assessing the doctor-patient relationship: different models or concepts are used. J Clin Epidemiol. (2012) 65:10–15. 10.1016/j.jclinepi.2011.05.011 22118265

[16] Harris M Guy D Picchio CA White TM Rhodes T Lazarus JV. Conceptualising hepatitis C stigma: A thematic synthesis of qualitative research. Int J Drug Policy. (2021) 96:103320. 10.1016/j.drugpo.2021.103320 34261587

[17] Talal AH Sofikitou EM Jaanimägi U Zeremski M Tobin JN Markatou M. A framework for patient-centered telemedicine: application and lessons learned from vulnerable populations. J Biomed Inform. (2020) 112:103622. 10.1016/j.jbi.2020.103622 33186707

[18] Islam MM. Missed opportunities for hepatitis C testing and other opportunistic health care. Am J Public Health. (2013) 103:e6. 10.2105/AJPH.2013.301611 24134357 PMC3828991

[19] Earnshaw V Smith L Copenhaver M. Drug addiction stigma in the context of methadone maintenance therapy: an investigation into understudied sources of stigma. Int J Mental Health Addict. (2013) 11:110–22. 10.1007/s11469-012-9402-5 23956702 PMC3743126

[20] Outland BE Erickson S Doherty R Fox W Ward L. Reforming physician payments to achieve greater equity and value in health care: a position paper of the American college of physicians. Ann Internal Med. (2022) 2022:4484. 10.7326/M21-4484 35724380

[21] Thomas-Henkel C Schulman M. Screening for Social Determinants of Health in Populations with Complex Needs: Implementation Considerations-Center for Health Care Strategies (2017). Available online at: http://www.chcs.org/resource/screening-social-determinants-health-populations-complex-needs-implementation-considerations/ (accessed on October 20, 2022).

[22] Taylor LA Tan AX Coyle CE Ndumele C Rogan E Canavan M. Leveraging the social determinants of health: what works? PLoS ONE. (2016) 11:e0160217. 10.1371/journal.pone.0160217 27532336 PMC4988629

[23] Zhang Y Li J Yu J Braun RT Casalino LP. Social determinants of health and geographic variation in medicare per beneficiary spending. JAMA Network Open. (2021) 4:e2113212-e2113212. 10.1001/jamanetworkopen.2021.13212 34110394 PMC8193453

[24] National Academies of Science. Reproducibility and Replicability in Science. Washington, DC: National Academy Press (2019). 31596559

[25] Stupple A Singerman D Celi LA. The reproducibility crisis in the age of digital medicine. NPJ Digit Med. (2019) 2:2. 10.1038/s41746-019-0079-z 31304352 PMC6550262

[26] Ioannidis JP. Why most published research findings are false. PLoS Med. (2005) 2:e124. 10.1371/journal.pmed.0020124 16060722 PMC1182327

[27] Ioannidis JPA. Correction: why most published research findings are false. PLoS Med. (2022) 19:e1004085. 10.1371/journal.pmed.1004085 36007233 PMC9410711

[28] Meng XL. Reproducibility, replicability, and reliability. Harvard Data Sci Rev. (2020) 2:dbfce7f9. 10.1162/99608f92.dbfce7f9 PMC10869125 38362534

[29] Talal AH Jaanimägi U Davis K Bailey J Bauer BM Dharia A. Facilitating engagement of persons with opioid use disorder in treatment for hepatitis C virus infection via telemedicine: stories of onsite case managers. J Substance Abuse Treat. (2021) 127:108421. 10.1016/j.jsat.2021.108421 34134875

[30] Talal AH Markatou M Sofikitou EM Brown LS Perumalswami P Dinani A. Patient-centered HCV care via telemedicine for individuals on medication for opioid use disorder: telemedicine for evaluation, adherence and medication for hepatitis C (TEAM-C). Contemporary Clin Trials. (2022) 112:106632. 10.1016/j.cct.2021.106632 34813962

[31] Brown AW Kaiser KA Allison DB. Issues with data and analyses: errors, underlying themes, and potential solutions. Proc Natl Acad Sci USA. (2018) 115:2563–70. 10.1073/pnas.1708279115 29531079 PMC5856502

[32] McShane LM Radmacher MD Freidlin B Yu R Li MC Simon R. Methods for assessing reproducibility of clustering patterns observed in analyses of microarray data. Bioinformatics. (2002) 18:1462–9. 10.1093/bioinformatics/18.11.1462 12424117

[33] Dolnicar S Leisch F. Evaluation of structure and reproducibility of cluster solutions using the bootstrap. Market Lett. (2010) 21:83–101. 10.1007/s11002-009-9083-420558260

[34] Bollon J Assale M Cina A Marangoni S Calabrese M Salvemini CB. Investigating how reproducibility and geometrical representation in UMAP dimensionality reduction impact the stratification of breast cancer tumors. Appl Sci. (2022) 12:4247. 10.3390/app12094247

[35] Kapp AV Tibshirani R. Are clusters found in one dataset present in another dataset? Biostatistics. (2006) 8:9–31. 10.1093/biostatistics/kxj029 16613834

[36] Brachmann M Spoth W Kennedy O Glavic B Mueller H Castelo S. Your notebook is not crumby enough, REPLace it. In: CIDR. Amsterdam (2020).

[37] Bethesda MD: My Own, Med, Inc. (2016). Available online at: https://myownmed.com/

[38] Wright A Andrews H Hutton B Dennis G. JSON schema: a media type for describing JSON documents. IETF Secretariat. (2022).

[39] Brachmann M Bautista C Castelo S Feng S Freire J Glavic B. Data debugging and exploration with vizier. In: SIGMOD-Demo. Amsterdam (2019).

[40] Yang Y Meneghetti N Fehling R Liu ZH Gawlick D Kennedy O. Lenses: an on-demand approach to ETL. pVLDB. (2015) 8:1578–89. 10.14778/2824032.2824055

[41] Crockford D Morningstar C. Standard ECMA-404 the JSON Data Interchange Syntax. Geneva: ECMA International (2017).

[42] Bray T. The JavaScript Object Notation (JSON) Data Interchange Format (2014). Available online at: http://www.rfc-editor.org/rfc/rfc7159.txt

[43] Pezoa F Reutter JL Suarez F Ugarte M Vrgoč D. Foundations of JSON schema. In: Proceedings of the 25th International Conference on World Wide Web. International World Wide Web Conferences Steering Committee. Montreal, QC (2016). p. 263–73.

[44] RJSF Team,. uiSchema. Read The Docs (2022). Available online at: http://react-jsonschema-form.readthedocs.io/en/latest/api-reference/uiSchema/

[45] RJSF Team. React JSONSchema Form. GitHub (2022). Available online at: http://github.com/rjsf-team/react-jsonschema-form

[46] Huser V DeFalco FJ Schuemie M Ryan PB Shang N Velez M. Multisite evaluation of a data quality tool for patient-level clinical data sets. EGEMS. (2016) 4:1239. 10.13063/2327-9214.1239 28154833 PMC5226382

[47] Sentence-Transformers. Sentence-Transformers/All-Mpnet-Base-v2 Hugging Face (2021). Available online at: http://huggingface.co/sentence-transformers/all-mpnet-base-v2 (accessed on October 07, 2022.

[48] Sentence-Transformers. Pretrained Models–Sentence-Transformers documentation (2021). Available online at: http://www.sbert.net/docs/pretrained_models.html (accessed on October 07, 2022).

[49] Bouveyron C Brunet-Saumard C. Model-based clustering of high-dimensional data: a review. Comput Stat Data Anal. (2014) 71:52–78. 10.1016/j.csda.2012.12.008

[50] Costa G Ortale R. Document clustering meets topic modeling with word embeddings. In: Proceedings of the 2020 SIAM International Conference on Data Mining (SDM). Cincinnati, OH (2020). p. 244–52.

[51] Schindler M Fox O Rausch A. Clustering source code elements by semantic similarity using Wikipedia. In: Proceedings of the Fourth International Workshop on Realizing Artificial Intelligence Synergies in Software Engineering. RAISE '15. Florence: IEEE Press (2015). p. 13–18.

[52] Abuaiadah D. Using bisect K-means clustering technique in the analysis of arabic documents. ACM Trans Asian Low Resour Lang Inf Process. (2016) 15:809. 10.1145/2812809

[53] Abd Rahman N Abu Bakar Z Zulkefli NSS. Malay document clustering using complete linkage clustering technique with Cosine Coefficient. In: 2015 IEEE Conference on Open Systems (ICOS). Melaka: IEEE (2015). p. 103–7.

[54] Shehata S. A Wordnet-based semantic model for enhancing text clustering. In: 2009 IEEE International Conference on Data Mining Workshops. Miami, FL: IEEE (2009). p. 477–82.

[55] Gupta M Rajavat A. Comparison of algorithms for document clustering. In: 2014 International Conference on Computational Intelligence and Communication Networks. Bhopal (2014). p. 541–5.

[56] Pedregosa F Varoquaux G Gramfort A Michel V Thirion B Grisel O. Scikit-learn: machine learning in python. J Mach Learn Res. (2011) 12:2825–30.

[57] McInnes L Healy J Saul N Grossberger L. UMAP: uniform manifold approximation and projection. J Open Source Software. (2018) 3:861. 10.21105/joss.00861

[58] Roweis ST Saul LK. Nonlinear dimensionality reduction by locally linear embedding. Science. (2000) 290:2323–26. 10.1126/science.290.5500.2323 11125150

[59] Kim H Kim HK Cho S. Improving spherical k-means for document clustering: fast initialization, sparse centroid projection, and efficient cluster labeling. Expert Syst Appl. (2020) 150:113288. 10.1016/j.eswa.2020.113288

[60] *Soyclustering: Python Clustering Algorithm Library for Document Clustering* (2020). Available online at: http://github.com/lovit/clustering4docs (accessed on January 25, 2023).

[61] *PhenX Toolkit: About* (2022). Available online at: http://www.phenxtoolkit.org/about (accessed on October 15, 2022).

[62] *PhenX Toolkit: Collections* (2022). Available online at: http://www.phenxtoolkit.org/collections/sdoh (accessed on October 15, 2022).

[63] Han J Kamber M Pei J. 2-Getting to Know Your Data. In:Han J Kamber M Pei J, editors. Data Mining (Third Edition). third edition ed. The Morgan Kaufmann Series in Data Management Systems. Boston, MA: Morgan Kaufmann (2012). p. 39–82.

[64] Kumari P Brachmann M Kennedy O Feng S Glavic B. DataSense: display agnostic data documentation. In: CIDR. Amsterdam (2021).

[65] Niu X Arab B Gawlick D Liu ZH Krishnaswamy V Kennedy O. Provenance-aware versioned dataworkspaces. In: TaPP. Mclean (2016).

[66] The Project Jupyter Steering Council. Project Jupyter (2022). Available online at: https://jupyter.org/

[67] The Apache Foundation. Apache Zeppelin (2022). Available online at: https://zeppelin.apache.org/

[68] Pimentel JF Murta L Braganholo V Freire J. A large-scale study about quality and reproducibility of jupyter notebooks. In:Storey MD Adams B Haiduc S, editors. Proceedings of the 16th International Conference on Mining Software Repositories, MSR. 2019 26–27 May Montreal, QC: IEEE/ACM (2019). p. 507–17.

[69] VanderPlas J. Idea: Jupyter Notebooks Could Have a “Reproducibility Mode.” (2017). Available online at: http://twitter.com/jakevdp/status/935178916490223616

[70] Evers-Meltzer J. Enforce a Top-Down Order of Execution (2018). Available online at: http://github.com/jupyter/notebook/issues/3229

[71] Freire J Glavic B Kennedy O Mueller H. The exception that improves the rule. In: HILDA. San Francisco, CA (2016).

[72] Feng S Huber A Glavic B Kennedy O. Uncertainty annotated databases-a lightweight approach for approximating certain answers. In: Proceedings of the 44th International Conference on Management of Data (2019).

[73] Feng S Huber A Glavic B Kennedy O. Efficient uncertainty tracking for complex queries with attribute-level bounds. In: Proceedings of the 46th International Conference on Management of Data. Virtual (2021). p. 528–40.

[74] Penman-Aguilar A Talih M Huang D Moonesinghe R Bouye K Beckles G. Measurement of health disparities, health inequities, and social determinants of health to support the advancement of health equity. J Public Health Manag Pract. (2016) 22:373. 10.1097/PHH.0000000000000373 26599027 PMC5845853

[75] Paterson BL Backmund M Hirsch G Yim C. The depiction of stigmatization in research about hepatitis C. Int J Drug Policy. (2007) 18:364–73. 10.1016/j.drugpo.2007.02.004 17854724

[76] Marinho RT Barreira DP. Hepatitis C, stigma and cure. World J Gastroenterol. (2013) 19:6703. 10.3748/wjg.v19.i40.6703 24187444 PMC3812468

[77] Treloar C Rance J Backmund M. Understanding barriers to hepatitis C virus care and stigmatization from a social perspective. Clin Infect Dis. (2013) 57:S51–5. 10.1093/cid/cit263 23884066

[78] Werremeyer A Mosher S Eukel H Skoy E Steig J Frenzel O. Pharmacists' stigma toward patients engaged in opioid misuse: when “social distance” does not mean disease prevention. Substance Abuse. (2021) 42:919–26. 10.1080/08897077.2021.1900988 33750283

[79] McNeil SR. Understanding substance use stigma. J Soc Work Pract Addict. (2021) 21:83–96. 10.1080/1533256X.2021.1890904

[80] Bush M. Addressing the root cause: rising health care costs and social determinants of health. North Carolina Med J. (2018) 79:26–9. 10.18043/ncm.79.1.26 29439099

[81] Houlihan J Leffler S. Assessing and addressing social determinants of health: a key competency for succeeding in value-based care. Primary Care. (2019) 46:561–74. 10.1016/j.pop.2019.07.013 31655752

[82] Sulley S Ndanga M. Inpatient opioid use disorder and social determinants of health: a nationwide analysis of the national inpatient sample (2012-2014 and 2016-2017). Cureus. (2020). 12:e11311. 10.7759/cureus.11311 33282587 PMC7714736

[83] Degan TJ Kelly PJ Robinson LD Deane FP. Health literacy in substance use disorder treatment: a latent profile analysis. J Subst Abuse Treatment. (2019) 96:46–52. 10.1016/j.jsat.2018.10.009 30466548

[84] Dahlman D Ekefäll M Garpenhag L. Health literacy among Swedish patients in opioid substitution treatment: a mixed-methods study. Drug Alcohol Depend. (2020) 214:108186. 10.1016/j.drugalcdep.2020.108186 32721789

[85] Degan TJ Kelly PJ Robinson LD Deane FP Smith AM. Health literacy of people living with mental illness or substance use disorders: a systematic review. Early Intervent Psychiatry. (2021) 15:1454–69. 10.1111/eip.13090 33254279

[86] Weiss BD. Health literacy Patient Safety: Help Patients Understand (2007). Available online at: http://www.partnershiphp.org/Providers/HealthServices/Documents/Health%20Education/CandLToolKit/2%20Manual%20for%20Clinicians.pdf (accessed on October 20, 2022).

[87] Paradies Y Ben J Denson N Elias A Priest N Pieterse A. Racism as a determinant of health: a systematic review and meta-analysis. PLoS ONE. (2015) 10:e0138511. 10.1371/journal.pone.0138511 26398658 PMC4580597

[88] Serchen J Doherty R Atiq O Hilden D. A comprehensive policy framework to understand and address disparities and discrimination in health and health care: a policy paper from the american college of physicians. Ann Internal Med. (2021) 174:529–32. 10.7326/M20-7219 33428444

[89] Report From Maternal Mortality Review Ccommittees: A View Into Their Critical Role (2017). Available online at: http://www.cdcfoundation.org/sites/default/files/upload/pdf/MMRIAReport.pdf (accessed on October 21, 2022).

[90] Medicaid Medical Directors Have A Front Row Seat To The Maternal Mortality Crisis. Here's What They're Focused On | Health Affairs (2020). Available online at: http://www.healthaffairs.org/do/10.1377/forefront.20200226.167484/full/ (accessed on October 21, 2022).

[91] Kimes PK Liu Y Neil Hayes D Marron JS. Statistical significance for hierarchical clustering. Biometrics. (2017) 73:811–21. 10.1111/biom.12647 28099990 PMC5708128

